# A Comparison Study on Multidomain EEG Features for Sleep Stage Classification

**DOI:** 10.1155/2017/4574079

**Published:** 2017-11-05

**Authors:** Yu Zhang, Bei Wang, Jin Jing, Jian Zhang, Junzhong Zou, Masatoshi Nakamura

**Affiliations:** ^1^Key Laboratory of Advanced Control and Optimization for Chemical Processes, East China University of Science and Technology, Ministry of Education, Shanghai, China; ^2^Department of Automation, School of Information Science and Engineering, East China University of Science and Technology, Shanghai, China; ^3^Research Institute of Systems Control, Institute for Advanced Research and Education, Saga, Japan

## Abstract

Feature extraction from physiological signals of EEG (electroencephalogram) is an essential part for sleep staging. In this study, multidomain feature extraction was investigated based on time domain analysis, nonlinear analysis, and frequency domain analysis. Unlike the traditional feature calculation in time domain, a sequence merging method was developed as a preprocessing procedure. The objective is to eliminate the clutter waveform and highlight the characteristic waveform for further analysis. The numbers of the characteristic activities were extracted as the features from time domain. The contributions of features from different domains to the sleep stages were compared. The effectiveness was further analyzed by automatic sleep stage classification and compared with the visual inspection. The overnight clinical sleep EEG recordings of 3 patients after the treatment of Continuous Positive Airway Pressure (CPAP) were tested. The obtained results showed that the developed method can highlight the characteristic activity which is useful for both automatic sleep staging and visual inspection. Furthermore, it can be a training tool for better understanding the appearance of characteristic waveforms from raw sleep EEG which is mixed and complex in time domain.

## 1. Introduction

Sleep is a natural process of humans for recovering energy and body health. It is considered as a necessity of life for humans and animals and is essential to their physical and emotional wellbeing. Physiologically, evaluating the quality of sleep depends on many aspects, including the duration and composition of sleep [1–4].

Rechtschaffen and Kales (R&K) defined sleep scoring criteria according to the change in the physiological signals [5]. Although there are several modifications and many amendments have been made, R&K criteria are still regarded as golden criteria for sleep staging in clinical application. According to R&K criteria, sleep is categorized by wakefulness (awake), rapid eye movement (REM), and nonrapid eye movement (NREM). NREM is further divided into sleep stages 1, 2, 3, and 4. Additionally, stages 3 and 4 are often combined together and refer to deep sleep or slow wave sleep (SWS) [6]. Clinicians can figure out whether one subject has a full rest by analyzing his/her overnight sleep measurement of PSG (polysomnogram) and provide a treatment plan based on the sleep stage inspection.

However, manual EEG interpretation by clinicians is time-consuming and the results were mainly dependent on human subjective judgments to some extent. Automatic sleep staging methods have been developed as an assisting tool for visual inspection [7]. Generally, the automatic sleep staging process can be described by four procedures: data acquisition, preprocessing, feature extraction, and classification. Obviously, feature extraction is an important procedure in sleep staging since the appropriate feature parameters can dramatically improve the classification results.

Feature extraction can reduce the dimensionality of EEG data and processing time. Till now, many feature analysis methods have been investigated, which cover several domains. Originally, time domain features include average of amplitude, variance, maximum, minimum, zero-crossing numbers, skewness, and kurtosis [8]. For further study, the detrended fluctuation analysis (DFA) and visibility graph (VG) based on sequence connectivity were utilized to analyze EEG signals and achieved good results [9, 10]. After that, some improved methods displayed more powerful capabilities such as multifractal detrended fluctuation analysis (MF-DFA) and horizontal visibility graph (HVG) [11, 12]. Apart from the time domain, transformed domain parameters were also proven to be useful in EEG researches. Ronzhina et al. put forward a single channel EEG based scheme by employing power spectral density (PSD) of EEG signals [13]. Huang et al. employed short-time Fourier transform on two channels of forehead EEG signals [14]. Furthermore, application of chaos theory and nonlinear time-series methods gave a deep insight into the brain dynamics reflected by EEG signals [15]. The nonlinear analysis methods based on the data complexity, including correlation dimension, fractal dimension, largest Lyapunov entropy, approximate entropy, sample entropy, and permutation entropy, were utilized [16–21]. Based on previous studies including time domain and frequency domain methods, the wavelet transform theory was frequently used to investigate EEG signals. Inoue et al. utilized a modified wavelet transform to extract peak frequency in time series to analyze all-night EEG data [22]. Ahmed et al. detected sleep spindle in EEG data by combining the wavelet transform theory and Teager energy [23]. Nowadays, the theory of wavelet transform is still in the process of perfecting and improving. Hassan and Bhuiyan proposed a tunable Q factor wavelet transform theory based on discrete wavelet transform which can adaptively divide EEG signal into several subbands and calculate the feature parameters [24].

In recent years, researches on feature extraction have mainly focused on frequency domain and nonlinear algorithms. However, there have been few academic achievements related to time domain feature extraction except for DFA and VG recently. In fact, clinicians generally interpret EEG by observing the waveforms in the signal. Theoretically, time domain analysis truly has a strong basis. Some of the authors proposed a time domain EEG analysis method which is based on the merger of the increasing and decreasing sequences to detect interictal epileptiform discharges [25]. EEG signal can be considered as the summation of several characteristic rhythms. After sequence merging, the feature rhythms in EEG signals can be detected more easily.

In this study, a comparison study on multidomain EEG features was presented. The ultimate purpose was to investigate the effective feature extraction method for automatic sleep staging. The powers of certain frequency components were calculated as the characteristic features in frequency domain. Approximate entropy was selected as the parameter of nonlinear dynamics. Instead of traditional time domain features, the merger of the increasing and decreasing sequences in EEG time series was developed according to the characteristics of sleep EEG for feature extraction in time domain. After feature calculation and extraction, the linear discriminate analysis (LDA) was adopted for sleep stage classification. The obtained classification results were compared with the visual inspection by a qualified clinician. The performance of each feature extraction method was discussed and the feasibility of the developed time domain method was analyzed.

## 2. Methods

### 2.1. Materials

The sleep data investigated in this study was recorded at the Department of Clinical Physiology, Toranomon Hospital, Tokyo, Japan. Three patients with breathing disorder during sleep (Sleep Apnea Syndrome) participated. Their ages ranged from 36 to 60. All of the three patients were males. Their overnight sleeping data were recorded after the treatment of Continuous Positive Airway Pressure (CPAP) based on the polysomnographic (PSG) measurement. The procedures were explained in detail to all participants, and informed consent was obtained before recordings were made.

The PSG measurement in Toranomon Hospital included 4 EEG channels (C3/A2, C4/A1, O1/A2, and O2/A1), 2 electrooculogram (EOG) channels (LOC/A1 and ROC/A1), and 1 electromyogram (EMG) channel (chin-EMG). The sampling frequency of EEG and EOG is 100 Hz and the sampling frequency of EMG is 200 Hz. Overnight PSG recordings were divided into consecutive 30-second epochs. The PSG recordings were inspected by a qualified clinician. Visual inspection was utilized to evaluate the effectiveness of automatic sleep stage classification.

### 2.2. Frequency Domain

The main characteristic activities related to sleep states in EEG are *δ* rhythm, *θ* rhythm, *α* rhythm, and *β* rhythm. Meanwhile, the waveforms of different sleep periods are significantly different according to the amount of these four activities. The amount of *α* rhythm is dominant in awake stage before sleep, while *θ* rhythm appears instead of *α* rhythm from awake to light sleep. The large amount of *δ* rhythm is the characteristic for deep sleep. Hence, calculating the power of a certain frequency component is helpful to distinguish sleep stages.

According to the sampling rate of EEG, one 30-second epoch contains 3000 points of data. The epoch is further divided into six 5-second segments. For each segment, the EEG data is converted from time domain to frequency domain by 512-point Fast Fourier Transform (FFT). The ratio of the power of certain frequency activity is calculated for each segment. The obtained values of 6 segments are averaged as the feature in frequency domain for a 30-second epoch.

In Table 1, there are four features referring to the ratio of the power of *δ*, *θ*, *α*, and *β* activity. The subscripts of FR indicate the frequency band of *δ*: 0.5–2 Hz; *θ*: 2–7 Hz; *α*: 8–13 Hz; *β*: 13–30 Hz, while *T* is the total EEG frequency band of 0.5–30 Hz. The power of each frequency band is obtained after FFT and the ratio to the total EEG frequency band is calculated. FR_*δ*_, FR_*θ*_, and FR_*β*_ are the averaged values of C3-A2 and C4-A2 channels, while FR_*α*_ is that of O1-A2 and O2-A1 channels.

### 2.3. Nonlinear Dynamics

Approximate entropy calculation is an algorithm based on the complexity of sequences. It is a developed statistic quantifying regularity and complexity, which appears to have potential application in a wide variety of relatively short (greater than 100 points) and noisy time-series data [26]. The greater the probability of producing a new pattern, the higher the complexity of the sequence and the larger the corresponding approximate entropy. The calculation of approximate entropy is as follows.

(1) Set a one-dimensional time series *u*(*i*)  (*i* = 1,2, 3,…, *N*) with the length of *N* and reconstruct an *m*-dimensional vector *X*_*i*_, *i* = 1,2,…, *N* − *m* + 1, as

(1) $$\begin{array}{c} X_{i}=\left\{\;u\left(\;i\;\right),u\left(\;i+1\;\right),\ldots ,u\left(\;i+m-1\;\right)\;\right\}. \end{array}$$

(2) Calculate the distance between vectors *X*_*i*_ and *X*_*j*_ by

(2) dij=max⁡ui+j−uj+k,k=0,1,2,…,m−1,

where the maximum value of the difference between two corresponding elements is the distance.

(3) Set one threshold *r* which is generally between 0.1 and 0.3. For each vector *X*_*i*_, add up the number of *d*_*ij*_ ≤ *r* × SD (SD is the standard value of the sequence) and calculate the ratio *C*_*i*_^*m*^(*r*) of the number to total distance (*n* − *m* + 1).

(4) Calculate ln⁡[*C*_*i*_^*m*^(*r*)] and obtain the average value *ϕ*^*m*^(*r*) by

(3) $$\begin{array}{c} \phi^{m}\left(\;r\;\right)=\frac{1}{N-m+1}\overset{N-m+1}{\underset{i=1}{\sum }}\ln C_{i}^{m}\left(\;r\;\right). \end{array}$$

(5) Increase *m* by 1 and repeat the steps from (1) to (4) to obtain *C*_*i*_^*m*+1^(*r*) and *ϕ*^*m*+1^(*r*).

(6) Calculate the approximate entropy value by

(4) $$\begin{array}{c} \text{ApEn}=\overset{}{\underset{N\to \infty }{\sum }}\left[\;\phi^{m}-\phi^{m+1}\;\right]. \end{array}$$

In this study, *r* represents the filtering level and *m* represents the length of run of data. These two parameters were set as 0.15 and 2, respectively. The averaged value of ApEn calculated from four recorded EEG channels was considered as the extracted feature by nonlinear analysis.

### 2.4. Time Domain

#### 2.4.1. Preprocessing

**Figure 1:**
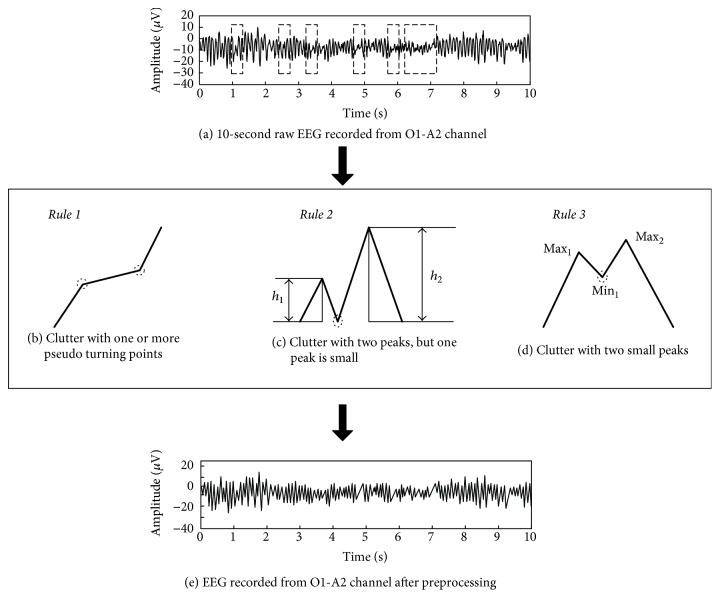
Preprocessing procedures before feature extraction from the time domain based on the sequence merging rules to eliminate the clutter from raw EEG.

As a general rule, a qualified clinician inspected EEG mainly based on the characteristic waveforms in sleep recordings.  Figure 1(a) gives a 10-second EEG signal from O1-A2 channel. It was inspected as one part of the awakening stage due to the large proportion of *α* rhythm. However, as shown in Figure 1(a), the sequences in boxes are generally seen as incomplete waveforms. These incomplete waveforms can be intelligently merged into the feature rhythm by experienced clinicians during EEG interpretation. This is the feature rhythm of *α* activity in the boxed sequences in Figure 1(a).

In order to intelligently interpret sleep EEG like humans, three rules were defined to simulate the process of merging those incomplete sequences as the clinicians. The defined rules are regarded as the preprocessing procedures before feature extraction in time domain. The objective is to eliminate the clutter from the raw EEG and enhance the feature rhythm for time domain analysis.

**Rule 1 .**

Figure 1(b) shows one kind of clutter. One or more pseudo turning points in the dotted circle can be observed. These pseudo turning points did not change the overall trend of the sequence and need to be eliminated. *y*_*h*_ is the *h*th sampling point in the sequence *y*. If *y*_*h*_ > *y*_*h*+1_ and *y*_*h*_ > *y*_*h*−1_, *y*_*h*_ is recognized as the local maximum in the sequence. Similarly, if *y*_*h*_ < *y*_*h*+1_ and *y*_*h*_ < *y*_*h*−1_ are satisfied, *y*_*h*_ is seen as the local minimum of the sequence. In this study, all of the maxima and minima of the signal are extracted to form a new time sequence. Finally, the turning points in the dotted circles are removed.

**Rule 2 .**

Figure 1(c) illustrates the second kind of clutter. The sequence contains two peaks. However, the amplitude of one of them is obviously small. The small peak is often seen as a pseudo peak during human interpretation. As shown in Figure 1(c), for two adjacent peaks, *h*_1_ and *h*_2_ are the two amplitudes of the two peaks. If *h*_1_/*h*_2_ ≤ 0.4 or *h*_2_/*h*_1_ ≥ 2.5, the peak with small amplitude is seen as a pseudo waveform. After the trough between the two peaks is removed as shown in the dotted circle in Figure 1(c), the second kind of clutter can be eliminated from the raw EEG.

**Rule 3 .**

Figure 1(d) shows the third kind of clutter. The sequence contained two peaks. The amplitudes of both peaks are small, and the point in the circle is often seen as a pseudo trough during human interpretation. In the research of detecting interictal epileptiform discharges, a method which is similar to Rule 2 was used to deal with this kind of clutter. However, considering that the amplitude of sleep EEG is different from epileptic EEG, the processing rule is modified as in Figure 1(d). As shown in Figure 1(d), point Max_1_ and point Max_2_ represent two peaks of sequence, and Min_1_ means the trough of the sequence. If Max_1_ − Min_1_ < 5 *μ*v and Max_2_ − Min_1_ < 5 *μ*v, the point Min_1_ is seen as a pseudo trough. Remove the pseudo trough and use Rule 1 to smooth the sequence.

The final processed EEG is shown in Figure 1(e). The clutter was removed after using the three rules mentioned above. Compared with the EEG signals in Figures 1(a) and 1(e), the characteristic waveforms became obvious, which is easy for both visual inspection and automatic analysis.

#### 2.4.2. Feature Extraction

After eliminating the clutter, the time domain parameters are calculated for classification. For example, as shown in Figure 1(e), the processed EEG signal has 10 real peaks in second 1. According to the definition of *α* rhythm (8–13 Hz), one *α* rhythm wave has 8–13 peaks in one second. Therefore, it is obvious that one *α* rhythm wave appeared in second 1. Similarly, there are 10 peaks in second 2, 9 peaks in second 3, 9 peaks in second 4, 9 peaks in second 5, and so on. Finally, the processed 10-second EEG signal in Figure 1(e) contains 10 *α* rhythmic waveforms, but no *δ*, *θ*, and *β* rhythmic waveform.

The numbers of the four rhythmic waveforms are counted as the features in time domain.  Table 2 illustrates the feature definition. The subscripts of TN indicate the frequency band of *δ*: 0.5–2 Hz; *θ*: 2–7 Hz; *α*: 8–13 Hz; *β*: 13–30 Hz. Each 30-second epoch is analyzed by the presented procedures. The number of each frequency band is counted after preprocessing. TN_*δ*_, TN_*θ*_, and TN_*β*_ are the averaged values of C3-A2 and C4-A2 channels, while TN_*α*_ is of O1-A2 and O2-A1.

### 2.5. Additional Features of EOG and EMG

Apart from the EEG features, electrooculogram (EOG) and electromyography (EMG) signals provide additional essential information for sleep staging. For example, the EEG pattern in REM stage is a mixed frequency activity which may be similar to adjacent stages. However, rapid eye movements can be observed in EOG, and EMG showed the lowest amplitude which is distinctive compared with the other sleep stages.  Table 3 illustrates the additional features of EOG and EMG from the time domain. The mean, variance, and span values are calculated from the two recording channels of EOG (LOC-A1 and ROC-A1). The zero-crossing number is obtained from chin-EMG. AM, AV, and AS are the averaged values of LOC-A1 and ROC-A1 channels. AZ is the zero-crossing value for chin-EMG.

## 3. Results

### 3.1. Feature Extraction

**Figure 2:**
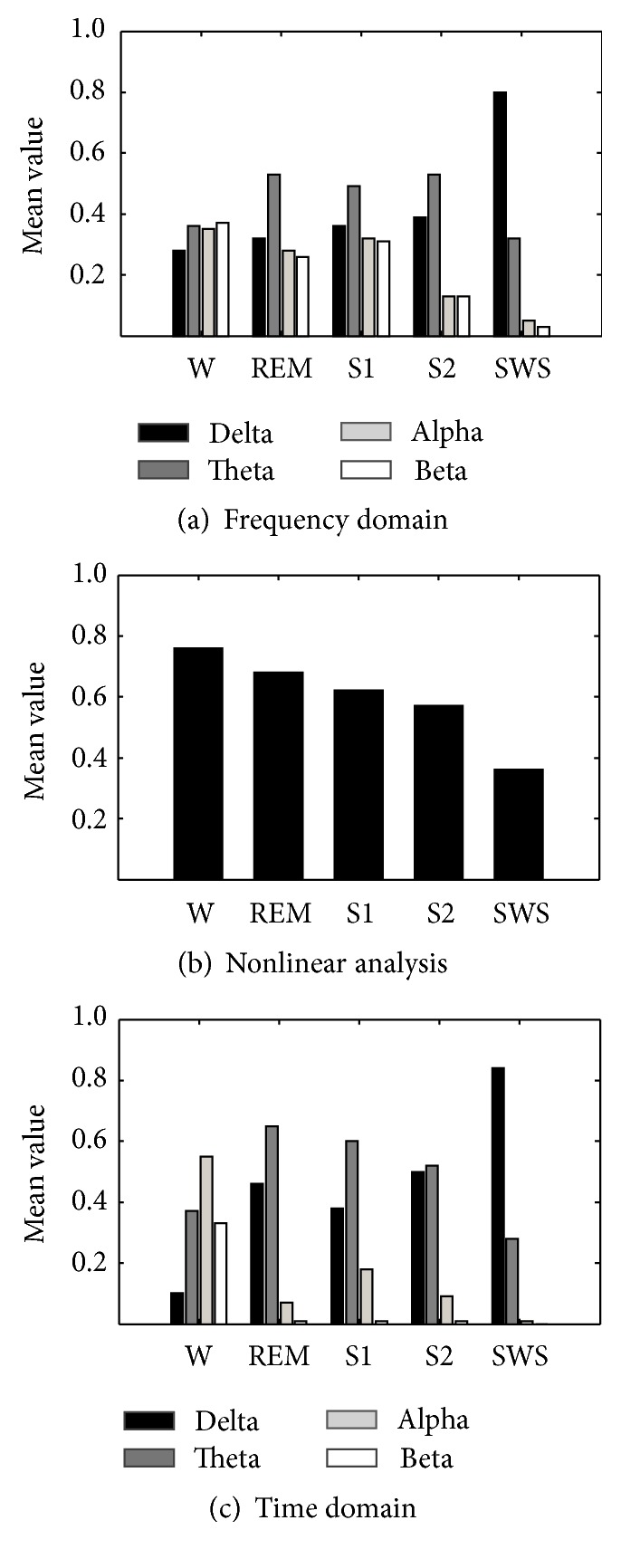
Comparison of the mean values of extracted features from frequency domain, nonlinear dynamics, and time domain among the sleep stages.

The overnight sleep recordings were analyzed. Mainly, there are 9 features extracted from the EEG signals, with 4 additional features from the EOG and chin-EMG. The extracted features were normalized in order to reduce the individual differences in EEGs.  Figure 2 illustrates the overall tendencies of EEG features of subject 1. The horizontal axis in Figure 2 represents sleep stages of awake stage, REM, sleep stage 1, sleep stage 2, and slow wave sleep. The vertical axis represents the mean value of each feature. In Figure 2, (a) indicates the features in frequency domain while (b) shows the features extracted by nonlinear analysis method and (c) shows the features from time domain. In Figures 2(a) and 2(c), the features related to *δ*, *θ*, and *β* rhythm were the average value of C3-A2 and C4-A1 channels, but *α* rhythm was of O2-A1 and O1-A2 channels. In Figure 2(b), the feature by nonlinear analysis was the average approximate entropy value of the four recording EEG channels.  Table 4 shows the statistical analysis results of each EEG feature for the three subjects, respectively. The numbers indicated the mean and variance of EEG features among the sleep states.

As shown in Figure 2(a), *δ* rhythm had a gradual increase with the depth of sleep. In addition, *θ* rhythm became quite dominant in REM, S1, and S2. In the awakening state, *α* rhythm and *β* rhythm occupied a large proportion. In general, *α* rhythm is dominant while the subject is relaxed and keeps his/her eyes closed. However, the phenomenon of “*α* rhythm blocking” will appear when the subject opens his/her eyes and *β* rhythm becomes obvious in EEG signals. Similar results can be observed from another two subjects in Table 4. The mean values of FR_*δ*_ of SWS and FR_*α*_ of W were higher than the other sleep states. The value of FR_*θ*_ showed that *θ* rhythm is more active in the light sleep states and rapid eye movement, while FR_*β*_ is in the awake stage. The overall tendency of feature values was consistent with the physiological knowledge.

Figure 2(b) shows the characteristics of nonlinear features with the changes of sleep stages. It is obvious that values of approximate entropy were decreasing with the depth of sleep. Theoretically, with the depth of sleep, the activity of the human brain gradually slows down. The data complexity of EEG signals varied with the changes of sleeping level. In Table 5, the ApEn values of three subjects were all gradually decreased from light sleep to deep sleep.

In Figure 2(c), the obtained variation trends of time domain features of subject 1 showed similar characteristics among the sleep stages when compared with those of the frequency domain. Similar results are illustrated in Table 4 for subject 2 and subject 3. Furthermore, the feature in time domain can highlight the characteristic of a certain EEG rhythm which can be obvious evidence for sleep stage classification. In the awake stage, *α* rhythm was apparently highlighted with the mean value of TN_*α*_ of 0.55, 0.57, and 0.41 for each subject in Table 4. This would be helpful for discriminating the awake stage from others. In the other cases, *δ* rhythm in slow wave stage of deep sleep was much more distinctive with the mean value of TN_*δ*_ of 0.84, 0.87, and 0.91. Comparing Figures 2(a) and 2(c), the extracted time domain features showed similar tendency among the sleep stages as well as the features in frequency domain. In addition, the features based on the developed sequence merging rules can highlight the characteristic rhythms in time domain. This would be easy for sleep stage scoring.

### 3.2. Classification Results

A linear discriminate analysis (LDA) classifier was adopted to compare the effectiveness of EEG features from different domains for automatic sleep stage scoring. The classification accuracy was calculated compared with the visual inspection in Table 5. Additionally, the number of consistent epochs within the total number of epochs was given under the accuracy value for each subject. The classification accuracy reached 87.09%, 83.11%, and 76.08% by using time domain features among the three subjects, respectively. The averaged accuracy in time domain was slightly higher than frequency domain and better than nonlinear method.

**Figure 3:**
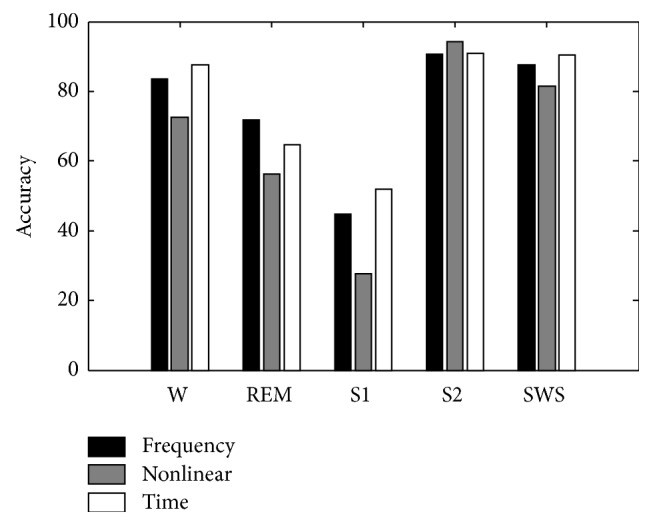
Comparison of classification accuracy of sleep stages.

Figure 3 showed detail evaluation about the classification results. The results were the average accuracies of the three test subjects for each sleep stage. It is apparent that both the time and the frequency domain features showed fairly good performance in REM and SWS, while the nonlinear algorithm performed the best in S2. Additionally, the features from the time domain also showed superiority in the recognition of awake and S1 stages.

## 4. Discussion

### 4.1. Frequency Domain and Nonlinear Analysis

The visual inspection of sleep recording required qualified ability and clinical experience. According to the recorded data, the four frequency activities of *δ* (0.5–2 Hz), *θ* (2–7 Hz), *α* (8–13 Hz), and *β* (13–30 Hz) almost covering the EEG frequency band were mainly inspected. Therefore, the amount of power of characteristic activities was commonly utilized as the feature extracted from the frequency domain for sleep stage classification. According to the evaluation results in Figure 3, features extracted from the frequency domain were able to provide rather good performance in sleep stage recognition.

During the overnight sleep process, the sleep levels were circled from light sleep to deep sleep about three times. When the sleep level was changed, the activities in sleep EEG were gradually changed accordingly. Approximate entropy refers to the complexity of the sequence. Through the obtained feature analysis result in Figure 2(b), the approximate entropy is well shaped to represent the change of the complexity of EEG signal from light sleep to deep sleep. In Figure 3, the automatic recognition result by approximate entropy had good performance to separate awakening and deep sleep. However, the accuracy of S1 was rather low, which was misclassified into S2 and REM.

### 4.2. Features Extracted from Time Domain

The features from frequency domain or by nonlinear analysis had merits for sleep stage classification. The limitation is also obvious for real clinical application. The frequency domain indicated the powers of certain characteristic activities. The differences between the sleep stages can be described by the change of power of those activities. However, the variation according to the time was missed. On the other hand, the traditional features from time domain can show the variation according to the time but not the characteristics in frequency domain as the clinician inspected.

The raw EEG can be regarded as the combination of characteristic activities including *δ*, *θ*, *α*, and *β*. In addition, it will inevitably be contaminated with various artifacts. In clinical practice, the qualified clinician had the skill to inspect the duration of the amount of characteristic activity by observing the original EEG time series. In this study, the feature extraction from time domain was developed in order to mimic the visual inspection. Before the feature extraction, a preprocessing procedure is proposed. There are three rules defined to eliminate the clutter and merge the EEG sequence to highlight the characteristic activities of *δ*, *θ*, *α*, and *β*. After preprocessing, the processed EEG signal can be used to easily inspect the characteristic waveforms in the sequences. According to the recognition results in Table 4 and Figure 3, the developed feature extraction method showed comparable performance to frequency domain and was better than the approximate entropy. It would be helpful to be an assisting tool for visual inspection. Furthermore, to a new or an unskilled technician, the presented feature extraction method can be a training tool for the clinicians to better understand the amount/duration of characteristic waveforms in sleep EEG. It may also be adopted as a training tool for such kind of users.

## 5. Conclusion

In this study, multidomain feature extraction was investigated for sleep EEG, including the amount of power of characteristic activity in frequency domain, the approximation entropy by nonlinear analysis, and the number of characteristic activities by a developed sequence merging method in time domain. Several features were extracted from sleep EOG and chin-EMG as additional parameters. The features of sleep EEG from different domains were analyzed and compared. The features from frequency domain showed consistent characteristics to the definition of sleep stage in criteria. The approximation entropy indicated a well gradually decreasing shape from light sleep to deep sleep. The features from time domain had similar tendency to the frequency domain. Furthermore, the corresponding characteristic activities can be highlighted compared with the frequency domain. Based on the features from different domains, the automatic sleep stage classification results were obtained and compared with the visual inspection. The classification accuracy in Table 4 and detailed comparison in Figure 3 indicated that the developed feature extraction method reached rather satisfying accuracy for sleep stage scoring than the frequency domain and nonlinear analysis.

The processed sleep EEG by the developed sequence merging method can highlight the characteristic rhythm which is useful for both automatic sleep staging and visual inspection. Furthermore, it can be a training tool for better understanding the appearance of characteristic waveforms from raw sleep EEG which is mixed and complex in time domain.

## Figures and Tables

**Table 1:** Features definitions in frequency domain.

Symbols	Notation	Equations
FR_*δ*_	Ratio of the power of *δ* activity in total EEG frequency band (%)	$ave\left\{\frac{S_{\delta }\left(C3\right)}{S_{T}\left(C3\right)}*100,\frac{S_{\delta }\left(C4\right)}{S_{T}\left(C4\right)}*100\right\}$
FR_*θ*_	Ratio of the power of *θ* activity in total EEG frequency band (%)	$ave\left\{\frac{S_{\theta }\left(C3\right)}{S_{T}\left(C3\right)}*100,\frac{S_{\theta }\left(C4\right)}{S_{T}\left(C4\right)}*100\right\}$
FR_*α*_	Ratio of the power of *α* activity in total EEG frequency band (%)	$ave\left\{\frac{S_{\alpha }\left(O1\right)}{S_{T}\left(O1\right)}*100,\frac{S_{\alpha }\left(O2\right)}{S_{T}\left(O2\right)}*100\right\}$
FR_*β*_	Ratio of the power of *β* activity in total EEG frequency band (%)	$ave\left\{\frac{S_{\beta }\left(C3\right)}{S_{T}\left(C3\right)}*100,\frac{S_{\beta }\left(C4\right)}{S_{T}\left(C4\right)}*100\right\}$

*δ*: 0.5–2 Hz; *θ*: 2–7 Hz; *α*: 8–13 Hz; *β*: 13–30 Hz; *T*: 0.5–30 Hz.

**Table 2:** Features definitions in time domain.

Symbols	Notation	Equations
TN_*δ*_	Number of *δ* rhythmic waveforms	ave⁡{TN_*δ*_(*C*3), TN_*δ*_(*C*4)}
TN_*θ*_	Number of *θ* rhythmic waveforms	ave⁡{TN_*θ*_(*C*3), TN_*θ*_(*C*4)}
TN_*α*_	Number of *α* rhythmic waveforms	ave⁡{TN_*α*_(*O*1), TN_*α*_(*O*2)}
TN_*β*_	Number of *β* rhythmic waveforms	ave⁡{TN_*β*_(*C*3), TN_*β*_(*C*4)}

*δ*: 0.5–2 Hz; *θ*: 2–7 Hz; *α*: 8–13 Hz; *β*: 13–30 Hz; *T*: 0.5–30 Hz.

**Table 3:** Additional features of EOG and EMG.

Symbols	Notation	Equations
AM	Mean value of EOG signal	ave⁡{AM(LOC), AM(ROC)}
AV	Variance value of EOG signal	ave⁡{AV(LOC), AV(ROC)}
AS	Span value of EOG signal	ave⁡{AS(LOC), AS(ROC)}
AC	Zero crossing of EMG signal	AC (chin-EMG)

**Table 4:** Feature extraction results.

Subject	State	FR_*δ*_	FR_*θ*_	FR_*α*_	FR_*β*_	ApEn	TN_*δ*_	TN_*θ*_	TN_*α*_	TN_*β*_
Subject 1	W	0.28 ± 0.12	0.36 ± 0.17	0.35 ± 0.15	0.37 ± 0.12	0.76 ± 0.10	0.10 ± 0.09	0.37 ± 0.16	0.55 ± 0.19	0.33 ± 0.13
	REM	0.32 ± 0.08	0.53 ± 0.09	0.28 ± 0.09	0.26 ± 0.06	0.68 ± 0.07	0.46 ± 0.08	0.65 ± 0.13	0.07 ± 0.04	0.01 ± 0.01
	S1	0.36 ± 0.11	0.49 ± 0.12	0.32 ± 0.10	0.31 ± 0.08	0.62 ± 0.05	0.38 ± 0.10	0.60 ± 0.12	0.18 ± 0.04	0.01 ± 0.01
	S2	0.39 ± 0.14	0.53 ± 0.13	0.13 ± 0.05	0.13 ± 0.06	0.57 ± 0.11	0.50 ± 0.10	0.52 ± 0.11	0.09 ± 0.04	0.01 ± 0.01
	SWS	0.80 ± 0.09	0.32 ± 0.09	0.05 ± 0.02	0.03 ± 0.01	0.36 ± 0.14	0.84 ± 0.07	0.28 ± 0.09	0.01 ± 0.01	0

Subject 2	W	0.30 ± 0.11	0.31 ± 0.11	0.52 ± 0.15	0.51 ± 0.18	0.78 ± 0.13	0.08 ± 0.06	0.41 ± 0.11	0.57 ± 0.12	0.22 ± 0.09
	REM	0.37 ± 0.13	0.68 ± 0.12	0.28 ± 0.08	0.33 ± 0.11	0.67 ± 0.08	0.49 ± 0.15	0.68 ± 0.14	0.05 ± 0.03	0.01 ± 0.01
	S1	0.31 ± 0.11	0.55 ± 0.11	0.43 ± 0.13	0.42 ± 0.11	0.72 ± 0.09	0.24 ± 0.10	0.75 ± 0.11	0.21 ± 0.08	0.03 ± 0.02
	S2	0.52 ± 0.15	0.63 ± 0.11	0.21 ± 0.07	0.25 ± 0.12	0.64 ± 0.12	0.45 ± 0.13	0.70 ± 0.14	0.09 ± 0.04	0.01 ± 0.01
	SWS	0.83 ± 0.06	0.37 ± 0.09	0.05 ± 0.03	0.02 ± 0.01	0.32 ± 0.11	0.87 ± 0.08	0.23 ± 0.09	0.01 ± 0.01	0

Subject 3	W	0.43 ± 0.18	0.43 ± 0.16	0.36 ± 0.16	0.47 ± 0.17	0.79 ± 0.18	0.11 ± 0.07	0.51 ± 0.11	0.41 ± 0.16	0.17 ± 0.09
	REM	0.30 ± 0.14	0.75 ± 0.16	0.29 ± 0.09	0.26 ± 0.11	0.75 ± 0.09	0.51 ± 0.14	0.66 ± 0.12	0.03 ± 0.01	0.01 ± 0.01
	S1	0.49 ± 0.14	0.54 ± 0.12	0.31 ± 0.17	0.37 ± 0.18	0.62 ± 0.18	0.38 ± 0.14	0.57 ± 0.12	0.17 ± 0.11	0.09 ± 0.05
	S2	0.49 ± 0.16	0.70 ± 0.17	0.19 ± 0.06	0.21 ± 0.08	0.56 ± 0.11	0.47 ± 0.11	0.54 ± 0.11	0.05 ± 0.03	0.02 ± 0.01
	SWS	0.82 ± 0.06	0.34 ± 0.09	0.03 ± 0.02	0.02 ± 0.01	0.33 ± 0.05	0.91 ± 0.06	0.20 ± 0.07	0.01 ± 0.01	0

**Table 5:** Comparison of classification results.

	Frequency domain + additional features	Nonlinear + additional features	Time domain + additional features
Subject 1	86.48% (851/984)	80.59% (793/984)	87.09% (857/984)
Subject 2	82.49% (811/983)	75.18% (739/983)	83.11% (817/983)
Subject 3	74.87% (704/940)	70.85% (666/940)	76.08% (715/940)
Average	81.38%	75.61%	82.18%
